# Comparison of Rapid to Standard Volumetric Modulated Arc Therapy for Palliative Radiotherapy in Lung Cancer Patients

**DOI:** 10.7759/cureus.10055

**Published:** 2020-08-26

**Authors:** David Y Mak, Ian Fraser, Robynn Ferris, Kerry James, Mitchell Liu, Steven D Thomas, Michael McKenzie, Shilo Lefresne

**Affiliations:** 1 Medicine, University of British Columbia, Vancouver, CAN; 2 Radiation Oncology, British Columbia Cancer Agency, Vancouver, CAN; 3 Medical Physics, British Columbia Cancer Agency, Vancouver, CAN

**Keywords:** palliative radiation, radiotherapy, lung cancer, vmat, rapid vmat, standard vmat

## Abstract

Patients with incurable lung cancer often present with debilitating symptoms that require urgent palliative radiotherapy. Volumetric modulated arc therapy (VMAT) provides several dosimetric advantages compared to basic non-conformal techniques, but involves complex planning resulting in a slower turn-around time for treatment. A simplified planning technique known as ‘rapid VMAT’ was developed with an aim to deliver palliative treatment to patients within 48 hours. The purpose of this study was to prospectively compare the dosimetric quality of rapid VMAT plans to standard VMAT plans. Fourteen consecutive rapid VMAT cases were re-planned de novo as per standard VMAT planning guidelines. Planning target volume (PTV) and organs at risk (OARs) were then compared. PTV coverage and dose to OARs including the spinal canal, lung, heart, and esophagus were similar between rapid and standard VMAT. Each plan was ready for treatment within 48 hours of the CT simulation. This study describes an expedited process for which palliative radiotherapy can be delivered to lung tumors with a similar robust quality that is provided for curative intent VMAT radiotherapy plans.

## Introduction

Lung cancer is the most commonly diagnosed cancer worldwide [1]. The majority of patients have advanced, incurable disease at diagnosis and often present with debilitating symptoms, such as dyspnea, pain, and hemoptysis, that frequently require urgent palliative radiotherapy [2,3]. Basic non-conformal radiotherapy techniques can allow for same-day radiotherapy planning and treatment but can result in neighbouring structures, such as the normal lung and esophagus, receiving a significant amount of the prescription dose resulting in acute toxicity that can impact quality of life [4].

As the use of volumetric modulated arc therapy (VMAT) becomes more widespread, interest in utilizing this advanced technique for treatments with palliative intent in an effort to decrease toxicity has grown [5-7]. While VMAT is associated with several dosimetric advantages that may translate into improved outcomes for patients, the technique involves complex planning and quality assurance that can strain department resources and result in slower turn-around times for patient treatments, which may often be unacceptable for symptomatic palliative patients. At our centre, the turnaround time for a ‘standard VMAT’ plan from date of CT simulation to treatment is typically 7-10 days. In an effort to increase capacity to provide timely VMAT to palliative lung cancer patients, a simplified planning technique known as ‘rapid VMAT’ was developed with an aim to deliver treatment to patients within 48 hours. The purpose of this study was to prospectively compare the dosimetric quality of rapid VMAT plans to standard VMAT plans for patients receiving palliative radiotherapy to lung tumors, and to assess its impact on the department workload.

## Materials and methods

Rapid VMAT development

**Figure 1:**
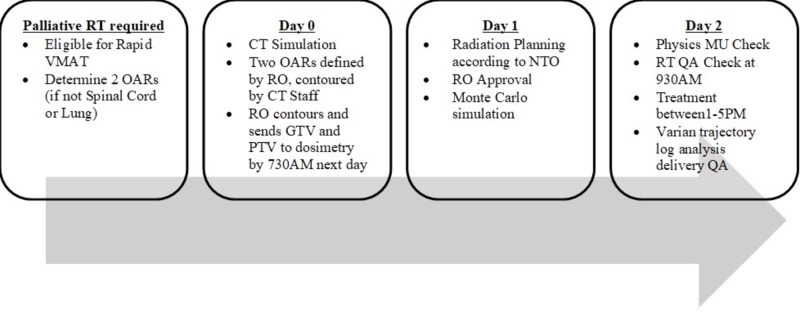
Rapid VMAT workflow VMAT: volumetric modulated arc therapy; RT: radiotherapy; OAR: organ at risk; RO: radiation oncologist; GTV: gross tumor volume; PTV: planning target volume; NTO: normal tissue objective; MU: monitor unit; QA: quality assurance

A committee of lung radiation oncologists (ROs), radiation therapists and physicists developed guidelines to describe patient eligibility, dose constraints, planning guidelines, and workflow for rapid VMAT with an aim to deliver treatment to patients within 48 hours of their CT simulation. Eligibility criteria and workflow are described in Table 1 and Figure 1.

**Table 1:** Eligibility criteria for rapid VMAT VMAT: volumetric modulated arc therapy; OAR: organ at risk

Rapid VMAT eligibility criteria
Radiation planning on non-contrast CT with no requirement for image registration
Only kV imaging for imaging verification
Radiation prescription in =<10 fractions
Maximum of dose constraints for 2 OARs
No pacemakers or hardware in the radiation field
No prior chest radiotherapy

 

Dose constraints for organs at risk (OARs) were adapted from constraints used for radical 30-fraction plans using the EQD2 formula with an α/β of 3. For example, instead of a lung V20 dose constraint for a 30-fraction plan, a V15 was requested for 10 fractions and V12 for 5 fraction plans. In an effort to decrease planning time, ROs were limited to providing dose constraints for only two OARs. All patients were contoured and analyzed using radiation planning software from Varian Medical Systems (Palo Alto, CA), specifically progressive resolution optimizer (PRO v11.0.31) for VMAT plan optimization, and anisotropic analytical algorithms (AAA v11.0.31) for volume dose calculations. Dose prescription (20 Gy/5 or 30 Gy/10) and tumor laterality-specific planning protocols were created by physics, using the Varian software ‘Clinical Protocol Templates’. These new protocols contained the predetermined planning parameters (dose, number of arcs, arc length, collimator rotations, OAR optimization values, etc) necessary for optimization. Plans were optimized according to normal tissue objective (NTO) first. Only if the dose constraints were not met with the NTO were plans further optimized by the dosimetrist. Once the protocol-defined values were met, dosimetrists sent the plans to the RO for approval. Following RO approval, the dosimetrist would send the plan directly for Monte Carlo simulation and quality assurance. This simplified process aimed to decrease the requirement of consultations with ROs and physicists during planning. Ten sample CT scans were used to test the rapid VMAT technique. Each plan was reviewed for quality and safety and rapid VMAT guidelines were finalized. The technique was successfully offered to patients in October 2017.

Comparison of rapid VMAT to standard VMAT

Between January 2018 and January 2019, 14 consecutive rapid VMAT cases were identified for this prospective study. Rapid VMAT planning was delivered as per standard and as outlined in Figure 1. Following treatment delivery, a single investigator contoured the additional OARs omitted in the rapid VMAT plan. A second radiotherapy plan was created de novo according to standard VMAT planning guidelines. The plan was reviewed by an RO for acceptability as per standard practice. Dosimetric parameters to the planning target volume (PTV) and OARs were collected by an independent investigator and converted to EQD2 equivalents for comparison.

Basic patient, tumor and treatment characteristics were summarized with descriptive statistics. Dosimetric comparisons between plans were conducted with paired t-tests and chi-square tests for continuous and categorical variables, respectively. Statistical analysis was performed on SPSS v14.0 (SPSS Inc., Chicago, IL).

## Results

Basic patient, tumor and treatment characteristics of the 14 plans are described in Table 2. The majority of patients had non-small cell lung cancer. Two patients had a primary tumour (colon, breast) that had metastasized to the lung. Dosimetric parameters for rapid VMAT and standard VMAT plans are presented in Table 3.

**Table 2:** Patient demographics PTV: planning target volume; OAR: organ at risk

Characteristic	Patients (n=14)
Median age (range)	72 (55–87)
Male, n (%)	10 (72)
Histology	
Non-small cell lung cancer, n (%)	10 (72)
Small cell lung cancer, n (%)	2 (14)
Other (metastasis), n (%)	2 (14)
Radiation prescription	
30 Gy/10, n (%)	7 (50)
20 Gy/5, n (%)	7 (50)
Median PTV volume (range)	650 (149–1733 cc)
OARs requested	
Lung, n (%)	14 (100)
Spinal cord, n (%)	12 (86)
Heart, n (%)	2 (14)
Plans ready within 48 hours, n (%)	14 (100)

**Table 3:** Dosimetric data for rapid and standard VMAT plans Doses are expressed as EQD2 equivalents. PTV: planning target volume; Dmax: maximum dose

	Rapid VMAT	Standard VMAT	p-value
PTV V95 (%)	98.5	99.0	0.33
Global Dmax (Gy)	36.3	36.3	0.70
Spine Dmax (Gy)	15.6	16.4	0.34
Lung V15/10 fractions or V12/5 fractions (%)	15.3	16.2	0.26
Lung mean (Gy)	4.9	4.9	0.96
Lung V4.5 (%)	40.5	39.6	0.62
Heart Dmax (Gy)	35.3	34.6	0.001
Heart mean (Gy)	5.5	5.5	0.97
Heart V22/10 fractions or V17/5 fractions (%)	8.9	9.4	0.605
Esophagus Dmax (Gy)	32.0	32.3	0.05
Esophagus mean (Gy)	8.5	8.3	0.19

PTV coverage, global maximum dose (Dmax), and dose delivered to OARs were similar between rapid VMAT and standard VMAT plans apart from Dmax to the heart (rapid 35.2 Gy vs standard 34.6 Gy, p=0.001), although this is unlikely to be of clinical significance. Both techniques provided acceptable PTV coverage and respected the provided dose constraints for OARs apart from the lung V4.5 (V4.5<60%). One patient (Case J) had a lung V4.5 of 65% with rapid VMAT, but this was lowered to 56% with the standard VMAT technique. On review, this case had the second largest PTV of the series, measuring 1679 cc. Another patient (Case M) had a lung V4.5 of 69% that was lowered to 65% with standard VMAT but still did not meet the requested constraint of <60%. Although the PTV in this case was only 773 cc, it involved the bilateral hilum and therefore a V4.5 of less than 60% would be geometrically challenging to achieve.

## Discussion

Our study demonstrated that a rapid VMAT technique produced palliative radiotherapy plans for lung tumors of similar quality to standard VMAT with excellent tumor coverage, and low doses to normal tissues. Each plan was approved for treatment within 48 hours of the CT planning scan.

The majority of studies exploring VMAT in the palliative setting have compared the dosimetry created by standard simple conformal techniques to intensity modulated techniques. Fog et al., for example, demonstrated VMAT provided higher PTV conformality and a lower bowel and kidney dose for palliative spinal cord compression treatments [5]. For palliative lung treatments, Iqbal et al. described improved PTV coverage and homogeneity with VMAT compared to a parallel opposed pair. There was no difference in the dose delivered to OARs; however, these structures were not included in the optimization process [6]. Our planning technique allowed ROs to select constraints for two OARs, but did not compare the subsequent dosimetry to the standard parallel opposed pair as the dosimetric advantages were expected based on a prior planning study in the radical setting [8].

The wait-time associated with palliative VMAT planning is addressed by Linden et al., who developed a VMAT technique to treat vertebral metastases the same day as CT simulation [9]. While our technique delivers VMAT within 48 hours of CT simulation, as experience with the technique develops, decreasing wait-time targets will be an important goal. It is also important to note that the dosimetric advantages provided by VMAT have not been proven to translate into improved patient outcomes. However, a specific esophageal sparing planning technique for palliative treatment was able to decrease mean esophageal doses from 16 to 8 Gy that is expected to lead to decreased rates of esophagitis according to a normal tissue complication probability model. This planning study has laid the foundation for PROACTIVE, a phase III trial exploring patient reported esophagitis with a parallel opposed pair compared to VMAT. Interestingly, in our study, although ROs could select esophagus as one of the two OARs, a constraint for this structure was not provided for any of the 14 VMAT plans.

This study should be considered in the context of its strengths and limitations. The sample size was small; however, the cases formed a small prospective cohort thereby limiting bias. While satisfaction with the rapid VMAT technique is high and a decrease in workload and planning complexity is subjectively reported by our ROs, dosimetrists and physicists, this study does not objectively quantify the impact of the rapid technique on the department workload. In addition, while this study demonstrates equivalent dosimetry between rapid and standard VMAT planning, palliative response and patient-reported toxicity were not investigated. Despite these limitations, this study is a unique and complementary addition to the literature that describes an expedited process for which palliative radiotherapy can be delivered to lung tumors with a similar robust quality that is provided for curative intent radiotherapy plans.

## Conclusions

The rapid VMAT technique has become a valuable tool for providing urgent palliative radiotherapy to patients with incurable lung cancer. Although evidence that VMAT decreases acute toxicity or improves quality of life compared to simpler radiotherapy techniques is lacking in the literature, it still seems reasonable to optimize available technologies according to the ALARA (as low as reasonably achievable) principle when feasible. Through a cautious, team-based approach, we were able to simplify the resource intensive planning and complex quality assurance associated with standard VMAT plans intended for higher dose, curative-intent plans, to a practical, streamlined process for lower dose, palliative-intent plans. Future directions will include adapting the rapid VMAT technique for other anatomic sites and further streamlining the process in an effort to deliver VMAT treatments on the same day as the CT simulation.
